# Application of flipped classroom teaching method based on ADDIE concept in clinical teaching for neurology residents

**DOI:** 10.1186/s12909-024-05343-z

**Published:** 2024-04-03

**Authors:** Juan Zhang, Hong Chen, Xie Wang, Xiaofeng Huang, Daojun Xie

**Affiliations:** 1 Department of Neurology, The First Affiliated Hospital of Anhui University of Traditional Chinese Medicine, 117 Meishan Road, Hefei, Anhui China; 2 The First Clinical Medical College of Anhui University of Chinese Medicine, Hefei, China

**Keywords:** ADDIE teaching model, Flipped classroom, Standardized training for residents, Neurology, Mini-CEX

## Abstract

**Background:**

As an important medical personnel training system in China, standardized residency training plays an important role in enriching residents’ clinical experience, improving their ability to communicate with patients and their clinical expertise. The difficulty of teaching neurology lies in the fact that there are many types of diseases, complicated conditions, and strong specialisation, which puts higher requirements on residents’ independent learning ability, the cultivation of critical thinking, and the learning effect. Based on the concept of ADDIE (Analysis-Design-Development-Implementation-Evaluation), this study combines the theory and clinical practice of flipped classroom teaching method to evaluate the teaching effect, so as to provide a basis and reference for the implementation of flipped classroom in the future of neurology residency training teaching.

**Methods:**

The participants of the study were 90 neurology residents in standardised training in our hospital in the classes of 2019 and 2020. A total of 90 residents were divided into a control group and an observation group of 45 cases each using the random number table method. The control group used traditional teaching methods, including problem based learning (PBL), case-based learning (CBL), and lecture-based learning (LBL). The observation group adopted the flipped classroom teaching method based on the ADDIE teaching concept. A unified assessment of the learning outcomes of the residents was conducted before they left the department in the fourth week, including the assessment of theoretical and skill knowledge, the assessment of independent learning ability, the assessment of critical thinking ability, and the assessment of clinical practice ability. Finally, the overall quality of teaching was assessed.

**Results:**

The theoretical and clinical skills assessment scores achieved by the observation group were significantly higher than those of the control group, and the results were statistically significant (*P* < 0.001). The scores of independent learning ability and critical thinking ability of the observation group were better than those of the control group, showing statistically significant differences (*P* < 0.001). The observation group was better than the control group in all indicators in terms of Mini-Cex score (*P* < 0.05). In addition, the observation group had better teaching quality compared to the control group (*P* < 0.001).

**Conclusion:**

Based on the concept of ADDIE combined with flipped classroom teaching method can effectively improve the teaching effect of standardized training of neurology residents, and had a positive effect on the improvement of residents’ autonomous learning ability, critical thinking ability, theoretical knowledge and clinical comprehensive ability.

## Introduction

As an important medical education system, the standardized residency training system is of great significance in China’s clinical medical training system [1–2]. In order to continuously improve the clinical medical talent training system and build a talent training system with clinical medical characteristics, China began to implement the resident standardized training system in 2014. Under the standardized clinical teaching plan, residents can achieve the requirements and objectives of multidisciplinary training required by the primary professional title through rotational learning and clinical teaching evaluation among various departments [3]. The implementation of the system not only greatly improves the professional ability of clinical medical staff, but also effectively saves medical resources and costs. However, neurology diseases are relatively abstruse and complex, with many critical diseases and strong professionalism, which requires physicians to have better autonomous learning ability, richer knowledge reserve and clinical emergency problem-solving ability.

The ADDIE model consists of five components: analysis, design, development, implementation, and evaluation [4]. The ADDIE teaching theory, as a new type of teaching theory, focuses on the needs and goals of the students. It allows the teacher to be the decision maker for learning [5], to set and develop the necessary learning steps and to implement them effectively by analysing the main learning objectives of the students and taking into account the students’ own realities. Learning effectiveness is checked through appropriate clinical teaching practice sessions to assess whether the learning requirements have been met, and it helps students to enhance their understanding of the learning content. It not only improves the educator’s ability to teach, but most importantly, the effectiveness of the students’ learning is also improved. Gagne instructional design method is mainly composed of nine learning events, such as training attention, informing learner of objectives, stimulating recall of prior learning, presenting stimulus, and providing learning guidance [6]. Compared with Gagne teaching design method, ADDIE model teaching method has the advantages of simple steps and easy implementation, and is often used in medical education design. Lucia et al. [7] used ADDIE model to develop the basic life support course in the process of adult cardiac arrest related surgery. Under the guidance of this theory, it not only realized the technical innovation in cardiopulmonary resuscitation education and systematization, but also had important positive significance for medical education. Maya et al. [8] developed and implemented the covid-19 elective course for pediatric residents by using the idea of ADDIE teaching. As an effective teaching method, this course provides necessary disaster response and flexible education for pediatric residents. Therefore, the teaching concept plays an important role in medical education.

Flipped classroom [9] was first popularised in the United States, where people advocated homework to replace the classroom learning format, and has gradually been applied to the medical education business in recent years [10]. It is different from traditional teaching. As an emerging mode of teaching, it advocates a student-centred approach, whereby the teacher prepares teaching videos or materials through an online platform and sends the materials to the students in a uniform manner before the students arrange their own study plan and time [11–12]. Therefore, this model is not limited by time and place, and students can learn according to their own situation and their own speed. When encountering difficult points, students can also watch the video repeatedly, interact and discuss with other students, or organise the questions and feedback them to the teacher for one-by-one answers.

Therefore, the flipped classroom teaching method based on AddIE teaching concept can formulate and implement the corresponding learning and training plan in combination with the clinical teaching needs of standardized training of neurology residents and the actual situation at this stage, encourage students to independently arrange learning time, and give the initiative of learning to students, so as to overcome the disadvantages of tight classroom time, heavy tasks, and students’ inability to study and think deeply in traditional medical teaching, which has a positive effect on the cultivation of students’ autonomous learning ability, the formation of critical thinking ability, and the improvement of professional knowledge and clinical comprehensive ability. Mini-CEX (Mini clinical exercise assessment) is considered to be an effective method for evaluating the clinical ability and teaching function of residents [13]. In this study, the theoretical and technical knowledge, autonomous learning ability and critical thinking ability were evaluated and scored, and the clinical comprehensive ability of residents was evaluated by mini CEX method, so as to provide a comprehensive and objective evaluation for clinical teaching results. This study is an exploration of medical clinical education mode, in order to provide reference for clinical teaching mode of standardized training of residents.

## Materials and methods

### Study design

A prospective controlled experimental design of research was used in this study.

### Participants

The participants of the study were 90 residents of the classes of 2019 and 2020 participating in the standardized residency training in the Department of Neurology of our hospital. Random number table method was used to divide 90 residents into control group and observation group with 45 residents in each group. There were 21 males and 24 females in the control group, aged 23–28 (25.40 ± 2.78) years. The observation group consisted of 23 males and 22 females, aged 22–27 (24.37 ± 2.59) years. All subjects signed an informed consent form. By comparing the general data of the residents in both groups, the results suggested no statistical significance (*p* > 0.05).

### Training methods

Both groups of residents underwent a one-month standardized residency training in the Department of Neurology. During the training period, the instructors trained the residents according to the standardized residency training syllabus, which mainly included theoretical learning and skills operation. The two groups of teachers were.

randomly assigned and the quality of teaching was monitored by the department head.

### Control group

**Fig. 1:**
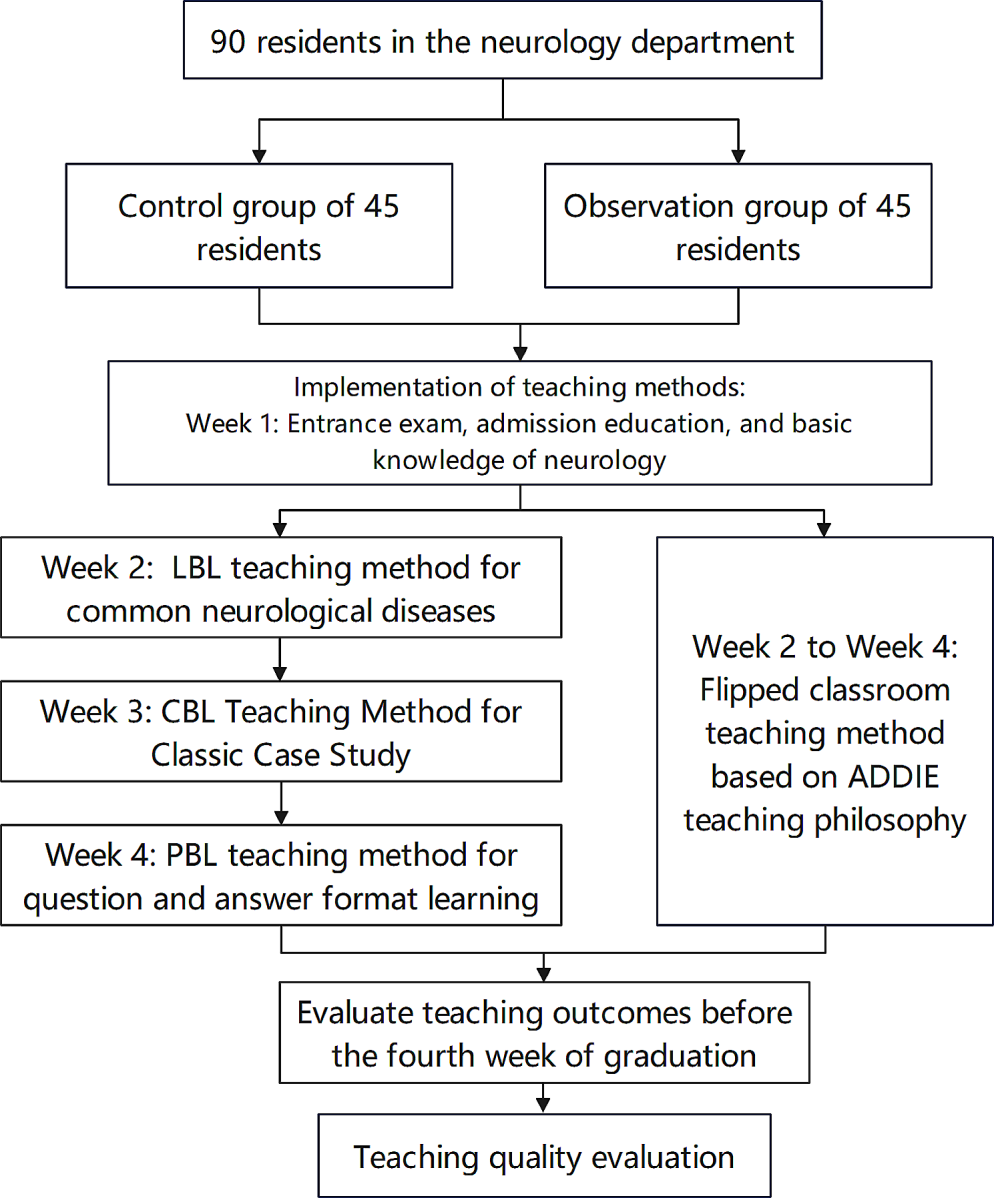
Flow chart of resident training process for two groups

The group adopted traditional teaching methods, including problem-based learning (PBL), case-based learning (CBL) and lecture based learning (LBL). PBL refers to a problem-oriented teaching method in which students seek solutions around problems [14]. CBL refers to the case-based teaching method, that is, to design cases according to teaching objectives, take teachers as the leading role, and let students think, analyze and discuss [15]. LBL refers to the traditional teaching method [16]. In the first week of enrollment, teachers will conduct unified enrollment assessment, enrollment education and popularization of basic knowledge of Neurology. The second week is mainly based on the traditional LBL teaching method, mainly for common diseases in the Department of Neurology, including ward round, bedside physical examination, auxiliary examination analysis, and putting forward the diagnosis basis and treatment plan. In the third week, CBL teaching method is mainly used to consolidate the knowledge learned through case study. In the fourth week, PBL teaching method is mainly used to promote problem learning and knowledge understanding by asking and answering questions. The learning outcomes were evaluated before leaving the department four weeks later. The detailed process was shown in Fig. 1.

### Observation group

This group adopted the flipped classroom teaching method based on the ADDIE teaching concept. The training content of the first week was the same as that of the control group. From the second to the fourth week, the flipped classroom teaching method based on the ADDIE teaching concept was adopted, with a total of 38 class hours. By analysing the content of the syllabus and the actual situation of the subjects, we designed and developed a characteristic and targeted teaching programme and implemented it, and conducted a unified assessment of the learning outcomes before the residents left the department in the fourth week. The concrete programme is shown in Table 1.

**Table 1:** Flipped classroom teaching plan based on ADDIE teaching concepts

Step	Teaching objectives	Teaching approach	Teaching evaluation	Class hours
Analysis	1. Complete the required teaching tasks. 2. Improve the overall clinical competence of residents.	Analysing and developing specific teaching programmes in combination with the actual learning situation of the students and the required teaching objectives.	1. Theoretical knowledge assessment. 2. Skills assessment. 3. Assessment of independent learning ability. 4. Critical thinking ability assessment.	4
Design	1. Promote residents’ self-directed learning skills. 2. Cultivate critical thinking of residents. 3. Improve communication and collaboration between residents and their teams.	1. Before the training, the teacher distributes theoretical knowledge and skills related to the course prepared in advance to the students in the form of texts or videos, etc. 2. Students learn through study materials and independent literature review. 3. Students discuss the difficulties encountered in the course through group work. 4. Teachers conduct bedside practical teaching.		4
Development	Development of specialised teaching methods based on the actual situation of the practitioner in order to improve the efficiency of learning.	Under the guidance of the teacher, the learning of theoretical knowledge will be further consolidated through the analysis of clinical teaching rooms and the allocation of specific learning tasks.		6
Imple mentation	1. Learning of professional theoretical knowledge. 2. Training of relevant clinical skills. 3. Cultivation of communication skills and enhancement of clinical humanistic care.	1. To sort out and report on special cases encountered in the clinic, and after the report, each group will analyse and discuss them, and continuously discuss and improve the treatment plan. 2. Teachers summarise and sort out the entire diagnosis and treatment process to promote students’ understanding and mastery of professional knowledge.		18
Evaluation	1. Cultivate personal clinical comprehensive abilities 2. Develop correct professional values	1. Students are assessed on their theoretical and professional skill knowledge at the end of the training. 2. Teachers comment and summarise the results of the assessment.	1. Teacher’s assessment of the student’s overall individual clinical competence 2. Assessment of teaching quality	6

### Step 1: composition of the teaching team

The members of the teaching team included a department head, 10 neurology lead teachers, and two non-neurology ADDIE specialists. The department chair is responsible for overseeing the overall quality of teaching, and the instructors are responsible for the teaching and learning of all students and the assessment of their outcomes. The ADDIE experts integrate the ADDIE concepts into the clinical learning curriculum plan of the standardised residency training according to the specific arrangement and actual situation of the curriculum.

### Step 2: setting of teaching objectives

The teaching objectives of standardised training for neurology residents mainly include the following aspects: (1) To understand and master common neurological diseases and their diagnosis and treatment processes, such as migraine, tension headache, benign paroxysmal positional vertigo, peripheral facial palsy, Parkinson’s disease, posterior circulation ischemia, cerebral infarction, cerebral hemorrhage, subarachnoid hemorrhage, epilepsy, etc.; (2) To understand and master systematic physical examination of the neurological system methods; (3) Proficiency in performing skillful operations related to neurological diseases, including lumbar puncture, etc.; (4) Familiarity with the management process of common neurological emergencies, including acute-phase cerebral infarction, acute-phase cerebral haemorrhage, and epileptic status persistent, etc.; and (5) Improvement of the resident’s ability of communicating with the team, collaborating with the team, communicating with the patients and the ability of dealing with the emergency problems on a temporary basis.

### Step 3: concrete teaching plan

With the unanimous agreement and unremitting efforts of the teaching team, the curriculum and methodology for the standardised training of residents in the flipped classroom based on the ADDIE teaching concept was finalised. The teaching plan will be carried out in 5 steps, as shown in Table 1.

### Step 4: implementation of flipped classroom teaching method based on ADDIE teaching philosophy

#### Project analysis

The final teaching task of this training mainly includes two aspects: (1) To complete all the teaching objectives set above; (2) To improve the residents’ comprehensive clinical ability in the process. Before the start of the training through the questionnaire form of the resident’s knowledge base of neurological specialities for the initial assessment, which helps to understand the current learning situation of the students, in order to facilitate the tailored teaching. At the same time, the main teaching tasks and teaching objectives were combined to analyse the specific form and content of the project, so as to develop a more practical and targeted programme.

#### Project design

The specific content of the project mainly includes: (1) Admission assessment: after admission to the department, all residents will conduct a unified admission mission and popularise the basic knowledge of neurology; (2) Flipped classroom teaching method: before the class, the leading teacher will analyse and sort out the common neurology diseases and their diagnosis and treatment processes according to the disease types based on the requirements of the syllabus, make a good teaching plan, and study a disease type at a time. Teachers will send teaching resources including PPT, video, cases, literature, etc. to the social platform. At the same time, they put forward the content and requirements to be mastered, and put forward 3–5 questions for students to think about in accordance with the focus of the teaching. Students can arrange their own study time, group themselves and have group discussions to try to solve the problems, and they can also ask questions to the teaching staff through the social platform at any time. Students can choose to go to the library or check the relevant literature on the Internet to expand their knowledge. In this session, knowledge transfer is completed; (3) Bedside practice teaching: the teacher communicates with the patient in advance, so that the students can conduct bedside questioning of medical history, physical examination, auxiliary examination and analysis. The diagnosis and diagnostic basis are proposed, and the teacher observes and assists the whole process.

#### Project development

After the teacher has finished the theoretical learning and practical teaching, he/she will ask targeted questions, pointing out what the students have done well and what needs to be improved in the process of questioning and treating the patients. At the same time, specific learning tasks are assigned for different students. Students are encouraged to report to the teacher about the patient’s condition and treatment plan, and propose their own treatment ideas. They are also allowed to ask the teacher any questions or problems that they cannot solve during the consultation. This teaching method is of great significance for students to master the theoretical knowledge of diseases and cultivate their clinical thinking.

#### Project implementation

Through the teaching team’s development of a specific and detailed teaching programme, methods such as entrance examination, flipped classroom teaching method, bedside practical teaching, and special case discussion were adopted. When encountering problems, students take the initiative to consult the literature and information or solve the problems independently through group discussion. If the problem cannot be solved, the students will seek help from the teachers, in order to practice students’ independent learning, teamwork and clinical diagnosis and treatment thinking ability.

#### Programme assessment

Students are assessed on their theoretical and professional skills knowledge at the end of the programme training. Students’ independent learning ability, critical thinking ability, clinical practice ability are assessed using relevant assessment methods, and finally the overall teaching quality is assessed, after which the teacher comments and summarises the results of the assessment.

### Observation indicators

#### Theory and skill knowledge assessment

This assessment includes two parts: theory and skill operation. The theoretical assessment mainly consists of the basic knowledge of neurology and the diagnosis and treatment process and medication of common neurology diseases. Skill operation involves lumbar puncture, thoracentesis, abdominal puncture, cardiopulmonary resuscitation, and other necessary items. The theory and skill operation parts were each worth 50 points, totalling 100 points. Unified assessment and grading will be conducted by the teachers.

#### Self-directed learning ability assessment scale

After the fourth week of training, the self-learning ability assessment form [17] was used to assess residents’ self-learning ability. The main contents include self motivation belief and objective behavior. Self motivation belief also includes self motivation (5 items) and learning belief (3 items). Objective behavior mainly includes four aspects: making learning goals and plans (4 items), self-monitoring and adjustment (7 items), obtaining and processing information (4 items) and communication and cooperation ability (7 items). The Likert scale [18] is used for a 5-level response system, which includes 5 levels of “completely non compliant”, “basically non compliant”, “average”, “basically compliant”, and “completely compliant”. The corresponding scores are 1 point, 2 point, 3 point, 4 point, and 5 point, with a total score of 150 points. The level of the score is positively correlated with the strength of autonomous learning ability. The Cronbach’s alpha coefficient was 0.929, the split half reliability was 0.892, and the content validity index was 0.970, indicating that the scale has good internal consistency, reliability and validity.

#### Critical thinking skills assessment scale

The Critical Thinking Skills Assessment Scale [19], which consists of seven dimensions, namely, truth-seeking, open-mindedness, analytical ability, and systematisation, with 10 items for each dimension, was used for the assessment at the end of the fourth week of training. A 6-point scale was used, ranging from “Strongly Disagree” to “Strongly Agree”, with scores ranging from 1 to 6, and the opposite for negative responses. The total score of the scale is 70–420, where ≤ 210 indicates negative performance, 211–279 indicates neutral performance, 280–349 indicates positive performance, and ≥ 350 indicates strong critical thinking skills. The Cronbach’s alpha coefficient was 0.90, the content validity index was 0.89, and the reliability was 0.90, indicating that the internal consistency, reliability and validity were good.

#### Clinical practice competence assessment

Clinical practice competence was assessed at the end of the fourth week of training using the mini-CEX scale [20], which included the following seven aspects: medical interview, physical examination, humanistic care, clinical diagnosis, communication skills, organisational effectiveness, and overall performance. Each aspect is rated from 1 to 9: 1 to 3 as “unqualified”; 4 to 6 as “qualified”; and 7 to 9 as “excellent”. The Cronbach’s alpha coefficient of the scale was 0.780, and the split-half reliability coefficient was 0.842, indicating that the internal consistency and reliability of the scale were relatively high.

#### Teaching quality assessment

Teaching quality assessment was conducted at the end of the fourth week of assessment, using the teaching quality assessment scale [21]. The specific content includes five aspects: teaching attitude, teaching method, teaching content, teaching characteristics, and teaching effect. The Likert 5-point scale was used, and the rating was positively correlated with the quality of teaching. The Cronbach’s alpha coefficient was 0.85 and the reliability was 0.83, which showed good reliability and validity.

### Data analysis

SPSS 23.0 statistical software was used to analyse the data. Measurement information was expressed as mean ± standard deviation ($\bar x \pm \,S$), and t-test was used for comparison between groups. Comparison of the unordered data between the two groups was performed using the χ2 test, or Fisher’s exact method. *p*-value < 0.05 was considered a statistically significant difference.

## Results

The scores and statistical analysis results of theory, skill assessment, self-learning ability assessment, critical thinking ability assessment of the two groups of students were shown in Table 2. The results of mini CEX assessment and statistical analysis were shown in Table 3. The results of teaching quality assessment and statistical analysis were shown in Table 4.

**Table 2:** Results of theory and skills assessment

	Number of subjects	Theoretical Assessment Scores	Skills Assessment Scores	Self-directed learning ability scores	Critical Thinking Skills Scores
Control group	45	41.94 ± 3.11	41.82 ± 3.54	102.65 ± 4.82	231.49 ± 10.73
Observation group	45	45.62 ± 2.83	46.76 ± 2.68	113.76 ± 4.31	239.38 ± 8.52
t-value		3.56	3.97	4.75	4.14
*p*-value		<0.001	<0.001	<0.001	<0.001

**Table 3:** Mini-CEX assessment results

	Control group	Observation group	t-value	*p*-value
Number of subjects	45	45		
Medical interviews	5.23 ± 1.21	6.57 ± 1.41	3.01	<0.01
Clinical examination	4.78 ± 1.49	5.89 ± 1.34	2.77	<0.01
Humanistic care	4.71 ± 1.42	5.56 ± 1.18	2.69	<0.05
Clinical diagnosis	5.36 ± 1.36	6.05 ± 1.53	2.35	<0.05
Communication skill	4.89 ± 1.04	5.48 ± 1.37	2.21	<0.05
Organisational effectiveness	5.14 ± 1.17	5.86 ± 1.62	2.47	<0.05
Overall performance	4.95 ± 1.25	5.77 ± 1.46	2.64	<0.05

**Table 4:** Results of teaching quality assessment

	Control group	Observation group	t-value	*p*-value
Number of subjects	45	45		
Teaching Quality Scores	91.04 ± 2.83	95.62 ± 2.31	3.62	<0.001

## Discussion

The standardised training of residents is an important medical personnel training system in China. It is a key link in the training of high-quality residents, which requires clinicians to have not only solid clinical expertise, but also noble medical character to better serve patients in outpatient and inpatient medical work. In recent years, due to the continuous development of China’s economic level, people’s demand for health is also increasing. Neurological system diseases are diverse, and certain diseases such as acute cerebrovascular disease, epilepsy, central nervous system infections, acute disseminated encephalomyelitis, Guillain-Barré, etc., have an acute onset and a rapid change in condition, which requires neurology residents to accurately identify and manage certain neurological emergencies and serious illnesses at an early stage. It puts forward higher requirements on the basic quality of neurology residents and brings more challenges to the clinical teaching of standardised neurology residency training. Therefore, the traditional teaching methods can no longer meet the current teaching requirements put forward under the new situation and new policies. Only by continuously improving and innovating the clinical teaching methods and improving the quality of teaching can the professional quality construction and training quality of residents be improved [22].

This study found that through four weeks’ teaching assessment, the theoretical and clinical skills assessment scores of the observation group were significantly higher than those of the control group, and the results were statistically significant (*P* < 0.001). Meanwhile, the scores of autonomous learning ability and critical thinking ability of the observation group were also better than those of the control group, with statistically significant differences (*P* < 0.001). In terms of Mini-Cex assessment, the observation group had better scores than the control group both in medical interview and physical examination (*P* < 0.01) and in humanistic care, clinical diagnosis, communication skills, organisational effectiveness, and overall performance (*P* < 0.05). In addition, the observation group also had higher scores compared to the control group regarding the quality of teaching in this study (*P* < 0.001). Previous studies have shown that the ADDIE concept can be applied to the design of clinical ethics education programmes and can be an effective tool for healthcare education, providing an established structure for the development of educational programmes [23]. Saeidnia [24] et al. used the ADDIE model to develop and design an educational application for COVID-19 self-prevention, self-care educational application to help people learn self-care skills at home during isolation, which can be used as an effective tool against COVID-19 to some extent. For the sake of reducing postoperative complications of breast cancer, Aydin [25] and others designed and developed a mobile application to support self-care of patients after breast cancer surgery with the support of the ADDIE model concept, which can provide professional medical guidance and advice for postoperative patients and is widely used in both education and clinical settings. Therefore, the ADDIE model concept has not only achieved better outcomes in the design of medical education, but also played a positive role in all aspects of disease prevention guidance and postoperative care.

As a flexible, targeted and effective new teaching method, flipped classroom method has been studied by many scholars in the field of basic medicine and clinical education. Pual [26] et al. found that the flipped classroom method was more effective for teaching clinical skills by comparing the two methods of course implementation, flipped teaching and online teaching. Du [27] and others found that a fully online flipped classroom approach increased classroom participation and adequate student-faculty interaction in distance education, and improved overall medical student exam pass rates during the COVID-19 pandemic, with better teaching and learning outcomes. Sierra [28] and others found that the flipped classroom method achieved better teaching and learning outcomes in a cardiology residency training programme, with higher acceptance among participants and teachers, and improved physicians’ assessment scores compared to traditional and virtual model teaching methods. Meanwhile, the Mini-CEX method was used in this study to assess the overall clinical competence of residents. This method, as a formative assessment, can not only provide a more accurate and comprehensive assessment of physicians’ comprehensive clinical competence, but also effectively promote physicians’ learning and growth [29–30]. Objective structured clinical examination(OSCE), as a method of evaluating students’ clinical comprehensive ability, understanding and application by simulating clinical scenarios, is widely used in the pre internship training of Undergraduates’ professional clinical practice skills [31]. Compared with OSCE, Mini-CEX is not limited by site and time, and it is time-consuming, simple and comprehensive. It can more systematically and comprehensively evaluate students’ clinical comprehensive ability [32–33]. Therefore, Mini-CEX is selected as the main clinical evaluation method in this study. Khalafi [34] et al. found that the use of Mini-CEX as a formative assessment method had a significant impact on the improvement of clinical skills of nursing anaesthesia students. Shafqat [35] et al. assessed the validity and feasibility of Mini-CEX by adopting it as a direct observation to assess its effectiveness and feasibility in an undergraduate medical curriculum. The study found that the altered method was effective in measuring student competence, improving clinical and diagnostic skills of medical students, and enhancing teacher-student interaction.

This study found that using ADDIE concept combined with flipped classroom teaching method, residents’ autonomous learning ability, critical thinking ability, theoretical knowledge and clinical comprehensive ability were improved. Analyze the potential causes: ADDIE, as a comprehensive medical teaching design concept, mainly includes five dimensions: analysis, design, development, implementation and evaluation. First, it systematically analyzes the specific clinical teaching needs and combines them with the current actual situation of students. On this basis, it flexibly sets the teaching plan, especially with the flipped classroom method, and pays attention to student-centered, This is quite different from the teacher centered concept in traditional teaching methods. This method encourages students to use their spare time to study independently through the text and video materials distributed by the teacher platform to meet the personalized needs of each student. At the same time, students actively explore the problems raised and encountered by teachers, which not only stimulate students’ interest in learning, but also greatly improve students’ autonomous learning and independent thinking ability. Furthermore, students’ collaborative discussion of problems and teachers’ in-depth explanation promoted the formation of students’ critical thinking, improved students’ learning effect and classroom efficiency, and improved students’ clinical comprehensive ability.

### Limitations and recommendations

Although this study achieved some clinical teaching value, we still have many shortcomings. First, the limited number of residency trainers resulted in an insufficient sample size for this study, which may have an impact on the results. Second, due to the limitations of the residency training syllabus and policy, the training in this study was conducted for only one month, in fact, the training of speciality knowledge and talent development often need more sufficient time. Third, the study only used the Mini-CEX to assess the residents’ comprehensive clinical competence, and the scale selection in this area is relatively homogeneous, which may have an impact on the real assessment results. Therefore, in the future, we will expand the sample size, giving more reasonable and sufficient time for teaching training and knowledge digestion and assimilation, by using multiple scales to conduct in-depth assessment in various aspects, with a view to obtaining more reliable and persuasive results, which will provide reference for the teaching of specialised clinical medicine.

## Conclusion

Based on the ADDIE concept combined with flipped classroom teaching method, this study conducted research in the residency training and found that compared with the traditional teaching method, the new teaching concept combined with flipped classroom teaching method can effectively improve the autonomous learning ability, critical thinking ability, theoretical knowledge and clinical comprehensive ability of neurology residents, and had better teaching quality. In clinical medical education, we should actively conform to modern teaching ideas. On the basis of traditional teaching, we should actively integrate new ideas and methods, give full play to the advantages of different teaching methods, so as to continuously improve the teaching efficiency and quality.

## Data Availability

The datasets used and/or analysed in this study are available from the corresponding author upon reasonable request.
